# Dorsal horn mapping: a Fiji toolbox for standardized laminar registration and transparent reporting of spinal cord imaging data from adult C57Bl/6 mice

**DOI:** 10.3389/fpain.2026.1961590

**Published:** 2026-10-07

**Authors:** L. Roettger, P. Adamek, S. G. Lechner

**Affiliations:** Department of Anesthesiology, University Medical Center Hamburg-Eppendorf, Hamburg, Germany

**Keywords:** dorsal horn, image analysis, image registration, rexed laminae, software, spinal cord

## Abstract

**Introduction:**

Rexed's laminae provide the anatomical framework for interpreting the spatial organization of neuronal populations within the spinal dorsal horn and are therefore widely used to describe immunohistochemical, *in situ* hybridization, and functional imaging data. However, despite their widespread use, no standardized approach for assigning cells to individual laminae has been established, limiting reproducibility and direct comparison of datasets across studies.

**Methods:**

We developed Dorsal Horn Mapping, an open-source Fiji toolbox that implements a standardized workflow for laminar registration in transverse mouse spinal cord sections. The toolbox aligns segment-specific reference maps to experimental images using user-guided rigid transformations and independent scaling, assigns labeled cells to Rexed's laminae, and transforms their positions into a common spatial reference frame. In addition, it provides tools for quantitative laminar analysis and visualization of pooled datasets as composite reference maps.

**Results:**

Validation experiments demonstrated high inter-user reproducibility of template alignment and laminar assignment across users with different levels of anatomical expertise. Registrations showed good agreement with established laminar markers and resulted in highly consistent assignment of neuronal populations to individual laminae. Thus, 81% of IB4-pos pixels were mapped to lamina II (95% CI: 68.4–86.2%) and the estimated difference in cell counts averaged across all users, segments and laminae was 0.08 cells (95% CI: −0.68 to 0.83; *P* = 0.843). Analysis of representative multichannel immunohistochemical datasets further demonstrated the utility of the workflow for standardized visualization and quantitative analysis of cellular localization.

**Conclusion:**

Dorsal Horn Mapping provides an accessible framework for standardized laminar registration and transparent spatial reporting of spinal cord imaging data from adult C57BL/6 mice. By introducing a common spatial reference system for dorsal horn anatomy, the toolbox facilitates direct comparison of cellular localization across experiments and laboratories and has the potential to improve reproducibility, comparability, and reporting standards in pain and spinal cord research.

## 1 Introduction

The mammalian spinal cord is a highly organized structure, both anatomically and functionally. It is divided into cervical, thoracic, lumbar, sacral, and coccygeal segments that correspond to the orderly emergence of spinal nerves. Within each segment, the gray matter is further subdivided into distinct cytoarchitectonic laminae, originally described by Rexed on the basis of neuronal morphology and cellular organization (1). Although this classification was initially anatomical, subsequent anatomical, electrophysiological, and molecular studies established that the individual laminae also represent functionally distinct domains with characteristic patterns of sensory input and local circuit organization (2–4). Consequently, Rexed's laminae have become the universal anatomical framework for describing the structural and functional organization of spinal sensory circuits.

Laminae I–VI, which together constitute the spinal dorsal horn, are of particular importance for somatosensory processing. Classical anatomical and electrophysiological studies demonstrated that different classes of primary sensory afferents terminate preferentially within distinct laminae. Nociceptive A*δ*- and C-fiber afferents project predominantly to the superficial dorsal horn (laminae I and II) (5–12), whereas low-threshold mechanoreceptors mainly innervate deeper laminae (III–V) (4, 13–16). Rather than acting as a passive relay station, the dorsal horn serves as the first central processing hub in which sensory information from different classes of primary afferents is integrated, filtered, and transformed before ascending signals are transmitted to higher brain regions, where the perceptions of touch and pain ultimately arise (17–19). Accordingly, the dorsal horn comprises numerous molecularly, morphologically, and functionally distinct neuronal populations, including projection neurons as well as excitatory and inhibitory interneurons. Recent advances in single-cell transcriptomics have further highlighted the remarkable cellular diversity of the dorsal horn by identifying dozens of transcriptionally distinct neuronal populations and providing increasingly comprehensive molecular atlases of spinal sensory circuits (20–22). Defining the spatial organization of these molecularly defined cell types and mapping the neural circuits they form has therefore become a central objective in contemporary spinal cord research.

Consequently, determining the laminar localization of neuronal populations has become a routine component of studies employing immunohistochemistry, *in situ* hybridization, activity-dependent labeling, viral tracing, calcium imaging, and, more recently, spatial transcriptomics. In most studies, the anatomical position of labeled cells is interpreted in the context of Rexed's laminae because laminar identity provides important information about the afferent input, local circuitry, and putative function of a given neuronal population. Despite the widespread use of Rexed's laminae as the anatomical reference system of the spinal dorsal horn, however, there is currently no broadly adopted standard for assigning experimental observations to individual laminae. Instead, laboratories typically rely on laboratory-specific workflows based on anatomical atlases, cytoarchitectonic landmarks, lamina-specific marker stainings, or manual estimation of laminar boundaries (16, 23–29). Consequently, the exact criteria used to define laminar borders are often incompletely described, making it difficult to directly compare datasets across studies and potentially contributing to inter-laboratory variability. As molecular atlases of the spinal cord continue to expand and increasingly large imaging datasets are generated, the need for standardized and reproducible spatial registration methods is becoming ever more pressing.

The registration of spinal cord sections is intrinsically challenging. Histological processing frequently introduces distortions through tissue shrinkage, stretching, compression, tearing, or partial tissue loss, while anatomical landmarks that clearly delineate all dorsal horn laminae are largely absent. Several approaches have therefore been developed to facilitate laminar identification. Manual delineation using anatomical atlases remains common practice, whereas others employ lamina-specific markers such as isolectin B4 (IB4), which labels non-peptidergic primary afferent terminals in inner lamina II (30, 31), or protein kinase C*γ* (PKC*γ*), which labels a characteristic band of interneurons concentrated around the transition between inner lamina II and lamina III (32, 33). Other studies estimate laminar boundaries based on predefined distances from the pial surface or use semi-automated registration approaches employing custom anatomical templates. While each of these strategies has distinct advantages, they also suffer from limitations, including observer dependence, restricted applicability to selected laminae, consumption of valuable fluorescence channels, limited transparency, or substantial manual effort. More importantly, because these approaches differ considerably between laboratories, they do not establish a common spatial reference system that facilitates direct comparison of independently acquired datasets.

More recently, Fiederling and colleagues introduced the SpinalJ framework, representing a major advance in standardized spinal cord image analysis (34, 35). By combining a three-dimensional spinal cord atlas with automated image registration, cell detection, atlas mapping, and quantitative spatial analysis, SpinalJ provides an exceptionally powerful platform for high-throughput analysis of spinal cord anatomy. However, the majority of spinal cord studies do not aim to reconstruct the entire spinal cord in three dimensions but instead analyze comparatively small numbers of transverse sections using conventional immunohistochemistry, *in situ* hybridization, or reporter mouse lines. For these applications, ease of use, transparency of the registration process, direct visual control of the alignment, minimal computational overhead, and straightforward integration into existing image analysis workflows are often more important than maximal registration flexibility or fully automated processing. Taken together, these considerations highlight the need for a practical registration framework that is standardized, transparent, readily accessible to non-specialist users, and sufficiently flexible for routine analysis of conventional spinal cord imaging datasets.

Here, we present “Dorsal Horn Mapping”, an open-source Fiji plugin that establishes a standardized workflow for laminar registration in transverse mouse spinal cord sections. The framework combines segment-specific dorsal horn reference maps covering spinal segments C1–L5 with user-guided image registration to generate normalized spatial coordinates within a common dorsal horn reference frame (Figure 1). This enables reproducible assignment of labeled cells to Rexed's laminae and visualization of complete datasets in a standardized anatomical context, thereby providing a substantially more transparent representation of spatial labeling patterns than isolated representative micrographs, which facilitates direct comparison of cellular localization across sections, animals, experimental conditions, and laboratories. Rather than replacing comprehensive atlas-registration platforms, our goal is to provide an accessible, reproducible, and open-source framework that lowers the technical barrier to standardized laminar registration and contributes to improved reproducibility, comparability, and reporting standards in spinal dorsal horn research.

**Figure 1:**
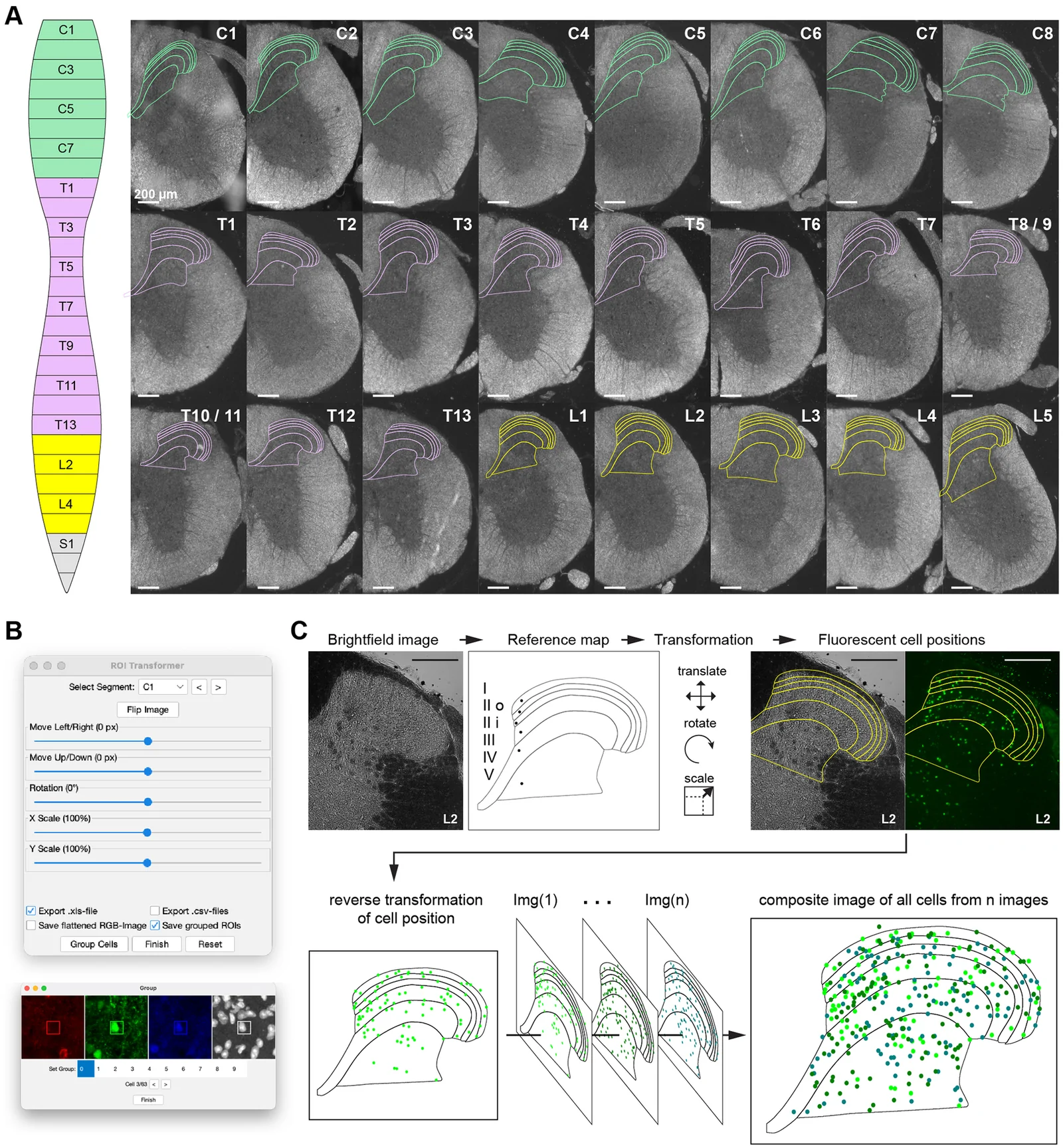
Overview of the plugin's functions and workflow. **(A)** Overlay of the reference maps generated for the segments from C1—L5; **(B)** Screenshots of the graphical user interface of the main window (top) and the ROI grouping feature (bottom). **(C)** Schematic representation of the workflow.

## 2 Materials and equipment

### 2.1 Software requirements

“Dorsal Horn Mapping” is implemented as an open-source plugin for Fiji (36). The plugin requires a current installation of Fiji running on Windows, macOS, or Linux together with a compatible Java Runtime Environment. The plugin has been successfully tested on macOS 12 and macOS 15 using Fiji version 2.16.0/1.54t with Java 1.8.0_322 (64-bit), as well as on Windows 11 using Fiji 1.54p and Java version 8. Future versions of the plugin are expected to remain compatible with newer Fiji releases. Fiji can be downloaded free of charge from the ImageJ project website.

The plugin is distributed through the official Fiji update mechanism and can therefore be installed directly from within Fiji without requiring manual compilation or installation of additional software packages. Source code, example datasets, and documentation are available through the project repository (see Data Availability Statement).

### 2.2 Hardware requirements

The plugin has no specialized hardware requirements and can be executed on standard desktop or laptop computers. We recommend a computer equipped with at least 8 GB RAM for comfortable handling of large fluorescence images, although substantially lower memory requirements are typically sufficient for routine analysis of individual spinal cord sections.

### 2.3 Input data

The workflow is designed for two-dimensional images of transverse mouse spinal cord sections acquired by brightfield or fluorescence microscopy. Image registration can be performed using either transmitted-light images or fluorescence images in which the boundary between gray and white matter is clearly discernible, for example by tissue autofluorescence. Cellular localization data are imported as ImageJ/Fiji regions of interest (ROIs), which may either be generated manually using Fiji's built-in selection tools or via automated image analysis workflows. Consequently, the toolbox can be readily integrated into existing image analysis pipelines irrespective of how the cells of interest were initially detected.

## 3 Methods

### 3.1 Overview of the workflow

The Dorsal Horn Mapping framework consists of three independent but complementary modules that together establish a standardized workflow for spatial analysis of transverse spinal cord sections (Figure 1). The first module performs image registration by aligning a segment-specific dorsal horn reference map to the experimental image. The second module transforms all labeled cells into normalized spatial coordinates within a common dorsal horn reference frame and assigns each cell to a Rexed lamina. Finally, the third module provides visualization tools that enable quantitative comparison of cellular distributions across images, animals, and experimental groups.

Although these modules are designed to function as an integrated analysis pipeline, they are independent of one another. Consequently, users may employ only selected components of the workflow. For example, transformed coordinates generated by the registration module can readily be imported into external software environments such as MATLAB or R for customized downstream analyses, while the plotting functions of the toolbox can likewise be applied to coordinate data generated by alternative image analysis pipelines.

The workflow is based on a simple but important principle. Rather than deforming experimental images to match an anatomical atlas, the reference map is interactively aligned to the image using rigid transformations and independent scaling in the horizontal and vertical dimensions. The resulting transformation parameters are subsequently applied in reverse to all labeled cells, thereby transforming their positions into normalized spatial coordinates within a common dorsal horn reference frame while leaving the original microscopy image completely unchanged. This approach preserves the integrity of the experimental data, allows every transformation to remain directly visible to the user throughout the registration process, and minimizes the risk of introducing image distortions that could arise from more complex nonlinear registration procedures. The complete workflow comprises four consecutive stages that are described in a step-by-step instruction below.

### 3.2 Spinal cord reference maps

Segment-specific dorsal horn reference maps were generated using the SC_P56_Atlas_10 × 10 × 20_v5_2020 dataset published by Fiederling and colleagues (34, 35), which has been created based on the Nissl stained spinal cord sections from the Allen Brain Atlas. For each spinal segment, the right dorsal horn was extracted from the corresponding atlas image and converted into a high-resolution vector representation using Inkscape. The boundaries of the individual Rexed laminae were manually traced, vectorized, and subsequently refined to generate continuous, non-overlapping anatomical regions suitable for image registration. Small discontinuities introduced during vectorization were corrected manually to ensure complete coverage of the dorsal horn without gaps between neighboring laminae. The finalized vector maps were imported into Fiji and converted into regions of interest (ROIs), thereby generating segment-specific anatomical templates covering spinal segments C1 to L5. Comparison of the atlas images revealed that the dorsal horn maps of segments Th8 and Th9 as well as Th10 and Th11 were identical within the original atlas. Consequently, these segments share a common reference map within the plugin. The origins of the reference coordinate systems were set to the arithmetic means of the points that constitute the dorsal horn outlines of the untransformed reference maps. The xy-coordinates of the laminar borders of the reference maps are provided in the Github repository for the Dorsal-Horn-Mapping plugin (see Data Availability Statement) in the folder …/Matlab_plotting/ROIOUTLINES/. Note, the shape of the dorsal horn changes along the rostro-caudal axis and therefore each segment has its own reference maps with its own coordinate system.

The resulting reference maps (Figure 1A) constitute the anatomical backbone of the registration framework and provide a standardized spatial reference system onto which experimental datasets from different animals, experiments, and laboratories can be projected.

### 3.3 Installation

Install the plugin directly via the FIJI update site. In FIJI's main window choose Help > Update and wait for the ImageJ Updater to open. Click “Manage Update Sites”, scroll down to “Dorsal Horn Mapping” and tick the box on the left. Click “Apply and Close” then click “Apply Changes” in the ImageJ Updater. After you restart FIJI, you will find the plugin under Plugins > Dorsal Horn Mapping.

### 3.4 Step-by-step user instruction

The following protocol describes the standard workflow for registering spinal cord sections, assigning labeled cells to Rexed's laminae, and generating normalized spatial coordinates for subsequent quantitative analysis.

#### 3.4.1 Step 1—open the image and load regions of interest

Open the spinal cord image to be analyzed in Fiji and load the corresponding ROI set containing the labeled cells. Alternatively, cells may be marked manually using any of Fiji's built-in selection tools (rectangle, oval, polygon, freehand, or point selections) and added to the ROI Manager by pressing “T”.

**Note:** At least one ROI must be present in the ROI Manager before the plugin can be executed. Users wishing to familiarize themselves with the workflow may instead load the example dataset provided with the plugin via “Plugins > Dorsal Horn Mapping > Example Dataset”.

#### 3.4.2 Step 2—start the plugin

Launch the registration interface by selecting “Plugins > Dorsal Horn Mapping > Mapping”, which will open the main registration window and display the controls required for image registration and data export (Figure 1B).

#### 3.4.3 Step 3—select the spinal segment

Choose the spinal segment corresponding to the analyzed tissue section from the drop-down menu at the top of the registration window.

If necessary, the selected segment can subsequently be changed either via the drop-down menu or the arrow buttons. Any transformations already applied to the reference map are automatically transferred to the newly selected segment. To restart the registration process, click “Reset”.

#### 3.4.4 Step 4—register the reference map

Align the segment-specific reference map to the dorsal horn visible in the microscopy image using the translation, rotation, and scaling controls (Figure 1C). Registration may be performed using either brightfield images or fluorescence images in which the boundary between gray and white matter is clearly discernible, for example through tissue autofluorescence. If required, use the “Flip Image” function to mirror both the image and the associated ROIs.

**Note:** Registration should be performed by aligning the outer contour of the dorsal horn gray matter rather than individual laminae or cellular landmarks. X- and y-scaling are applied to the original untransformed reference map such that the arithmetic mean of the map stays in the same position, and rotation is also performed around the arithmetic mean. Hence, when re-applying the transformation parameters (e.g., to reproduce previous results), the individual steps can be applied in any random order.

#### 3.4.5 Step 5—assign ROIs to experimental groups (optional)

For multichannel experiments, ROIs can be assigned to different experimental groups to distinguish individual cell populations. Group assignment may either be performed before starting the plugin using Fiji's built-in ROI grouping function or interactively within the plugin. To assign groups during the analysis, click “Group Cells”. A separate window will open displaying enlarged views of the current ROI in all image channels together with a selection bar containing ten possible groups (0–9). Cells can be assigned either by clicking the desired group number or by pressing the corresponding number key on the keyboard. Navigate between cells using the arrow buttons or the keyboard cursor keys. After all cells have been classified, click “Finish” to return to the main registration window (Figure 1B, bottom).

**Note:** Group assignments are preserved throughout the workflow and are used by the plotting module to visualize different cellular populations separately.

#### 3.4.6 Step 6—select export options

Before completing the analysis, choose the desired output files. The plugin can export (i) an Excel spreadsheet containing original image coordinates, normalized coordinates, laminar identity, spinal segment, and ROI group, (ii) comma-separated value (CSV) files containing the same information, (iii) a flattened PNG image showing the registered reference map together with all ROIs and (iv) grouped ROI sets preserving the assigned experimental groups. In addition, all transformation parameters applied during registration are exported to ensure complete documentation and reproducibility of the analysis.

#### 3.4.7 Step 7—perform the registration

Click “Finish” to complete the registration. The plugin transforms all ROIs into the common dorsal horn reference frame, assigns each ROI to its corresponding Rexed lamina, exports the selected output files, and displays a results table containing ROI identity, original image coordinates, normalized coordinates, laminar assignment, spinal segment, and experimental group.

#### 3.4.8 Step 8—analyze additional images

To analyze another section, open the corresponding image, load the associated ROI set, click “Reset”, and repeat the registration procedure beginning with step 3.

#### 3.4.9 Step 9—generate composite reference maps

To visualize pooled datasets, open the plotting interface by selecting “Plugins > Dorsal Horn Mapping > Plotter”. Next, select the folder containing the exported spreadsheets generated during Step 7. The plotting interface provides two visualization modes. Selecting “By Segment” mode combines all registered cells from one spinal segment into a single composite reference map, enabling direct comparison of pooled datasets across multiple sections (Figure 1C). Selecting “By Image” mode displays each analyzed section individually and is primarily intended for quality control, troubleshooting, and identification of potential outliers. The exported coordinate files can also be imported into external software environments, including MATLAB or R, enabling users to perform customized downstream analyses while retaining the standardized spatial reference system established by the registration workflow.

**Note:** The “Plotter” will only plot data that are provided in the Excel file format as it is saved by the registration tool. Any deletion or insertion of columns will lead to errors while plotting. The “Plotter” is intended to plot the data of several experiments at once and will thus always plot all available data inside the selected folder. To limit the plotted data, create a new folder and copy only the data you want to plot into it before running the “Plotter”.

### 3.5 Evaluation of reproducibility and robustness

A primary objective of our “Dorsal Horn Mapping” plugin is to establish a standardized and reproducible workflow for assigning labeled cells to Rexed's laminae. We therefore performed three complementary validation experiments to evaluate (i) the reproducibility of reference map registration, (ii) the anatomical accuracy of the resulting laminar alignment, and (iii) the robustness of downstream laminar cell assignment.

#### 3.5.1 Evaluation of inter-user reproducibility

To assess the reproducibility of the registration procedure, five independent users analyzed a total of 48 images comprising 24 brightfield images and 24 fluorescence images in which the gray and white matter could be readily distinguished by their intrinsic autofluorescence (Figure 2A and Supplementary Material for description of experimental procedure). The users represented different levels of prior experience, ranging from researchers with extensive expertise in spinal cord anatomy and physiology to technical staff without previous experience in spinal cord image analysis.

**Figure 2:**
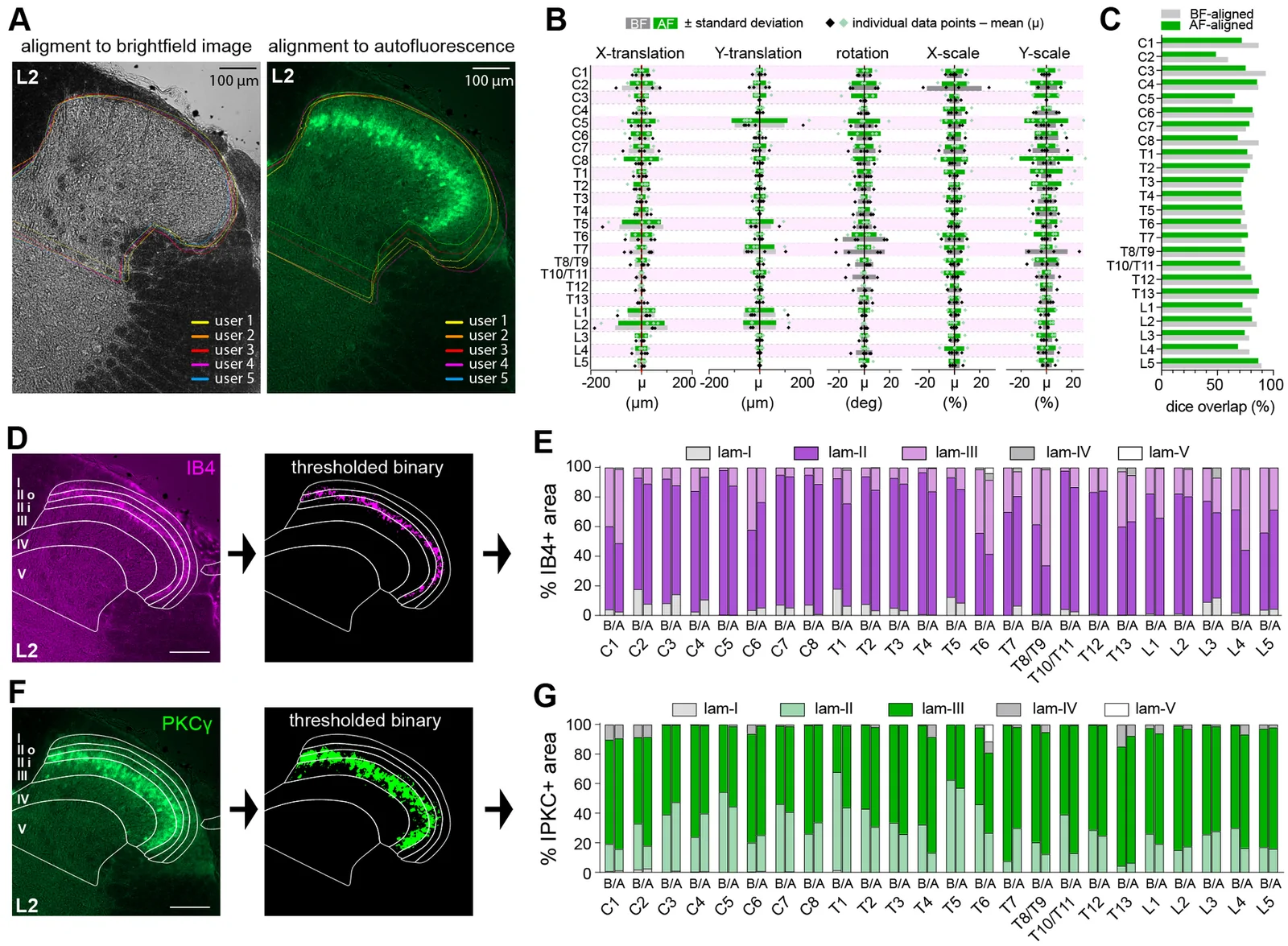
Evaluation of inter-user variability and comparison with established lamina markers. **(A)** Representative brightfield- and fluorescence image of the same L2 dorsal horn section with superimposed reference maps aligned by the indicated users. **(B)** Comparison of the standard deviations of the five transformation parameters applied by the five independent users during map alignment to brightfield (BF) and autofluorescence (AF) images. The horizontal bars show that standard deviations and the black diamond symbols the individual transformation parameters for each user. Note, for better comparison all data are shown as mean-centered values. **(C)** To compare the similarity of the final aligned reference maps, dice-overlap (i.e., the percentage of the area covered by all five user-aligned reference maps) was calculated and is shown for both BF-aligned (grey bars) and AF-aligned (green bars) maps. **(D)** Raw (left) and thresholded binary (right) image of IB4 staining of the L2 section show in A. **(E)** Comparison of the percentage of IB4-positive pixels in the thresholded binary across laminae I-V, for each spinal segment and both alignment modalities (left bar, brightfield-aligned **(B)** Right bar, autofluorescence-aligned **(A)**. **(F)** Raw (left) and thresholded binary (right) image of PKC*γ*-staining of the L2 section show in A. **(G)** Comparison of the percentage of IB4-positive pixels in the thresholded binary across laminae I-V, for each spinal segment and both alignment modalities (left bar, brightfield-aligned **(B)**, right bar, autofluorescence-aligned **(A)**.

Before starting the evaluation, all participants received a short introduction to the functionality of the plugin together with a brief overview of the anatomical organization of the spinal cord. No further instructions regarding image registration or template alignment were provided. Each participant was then asked to align the segment-specific reference maps to 24 predefined spinal cord sections spanning segments C1 to L5 using either the corresponding brightfield image or the autofluorescence image. Technical assistance regarding operation of the software was available throughout the evaluation, but no guidance concerning anatomical alignment was given.

For each registration, the plugin automatically exported the applied transformation parameters, including horizontal and vertical translation, rotation, and scaling in the x- and y-directions (Supplementary Tables 1, 2). Inter-user variability was quantified by calculating the standard deviation for each transformation parameter across all users, which are shown in Figure 2B as mean-centered horizontal bars. For brightfield-aligned reference maps standard deviations ranged from 10.7–103.5 µm (95% CI: 24.1–44.8) for x-translation, 1.96–97.6 µm (95% CI: 13.3–34.4) for y-translation, 1.2–16.9 deg (95% CI: 4.5–7.9) for rotation, 1.7–21.7% (95% CI: 3.3–6.6) for x-scale and 0–16.7% (95% CI: 4.9–7.9) for y-scale. For autofluorescence-aligned reference maps standard deviations ranged from 2.64–90.8 µm (95% CI: 23.8–42.8) for x-translation, 6.85–109.9 µm (95% CI: 16.49–37.47) for y-translation, 0.9–12.9 deg (95% CI: 4.1–7.1) for rotation, 3.6–10.6% (95% CI: 5.7–7.5) for x-scale and 3.3–21.2% (95% CI: 6.7–10.4) for y-scale. The transformation parameters applied by individual users are shown as symbols in Figure 2B, which reveals that particularly large variabilities were usually caused by the user with no prior knowledge about spinal cord anatomy and do therefore not indicate a general lack of reproducibility. Importantly we also analyzed the percentage of the area of the entire dorsal horn map covered by all five user-aligned reference maps for both alignment modalities (Figure 2C). This analysis showed that overall overlap was similar across all spinal segments regardless of the standard deviations of individual parameters, indicating that different combinations of the five transformation parameters can result in similar overall alignment. Across all segments the median overlap was 78.5% (95% CI: 74.6–85.3%) for brightfield-aligned maps and 74.4% (95% CI: 71.4–79.1%) for autofluorescence-aligned maps. The only exception was segment C2, which consistently exhibited small dice-overlap and large standard deviations in both alignment modalities, suggesting that this segment presents a greater challenge for manual registration than the remaining spinal levels.

#### 3.5.2 Validation using established laminar markers

We next examined whether registration based solely on the outline of the dorsal horn accurately reproduced the position of well-established anatomical landmarks. To this end, spinal cord sections were labeled with isolectin B4 (IB4), which prominently labels non-peptidergic primary afferent terminals within inner lamina II, and an antibody against protein kinase C*γ* (PKC*γ*), which labels neurons concentrated around the transition between inner lamina II and lamina III (see Supplementary Material for description of experimental procedure).

Reference maps that had previously been aligned to either the corresponding brightfield images or autofluorescence images (Figure 2A) were superimposed onto thresholded binary images of the IB4 and PKC*γ* labeling (Figures 2D,F). To quantify anatomical agreement, the fraction of IB4-positive and PKC*γ*-positive pixels located within each Rexed lamina was determined for every spinal segment (Figures 2E,G). This analysis showed that across all spinal segments the majority of IB4-pos pixels aligned with lamina II for both reference map alignment modalities (IIi + IIo, median across all spinal segments for brightfield-aligned maps: 81%, 95% CI: 68.4–86.2%; median across all spinal segments for autofluorescence-aligned maps: 75.5%, 95% CI: 65.9–83.1%; Figure 2E). Likewise, the majority of PKC*γ*-pos pixels were distributed across lamina Ili and III (brightfield-aligned median lamina-II = 29.3%, 95% CI: 20.5–39.3; brightfield-aligned median lamina-III = 69.9%, 95% CI: 58.5–74.1; autofluorescence-aligned median lamina-II = 25.5%, 95% CI: 16.2–33.8; autofluorescence -aligned median lamina-III = 73.7%, 95% CI: 59.6–76.9; Figure 2G). At this point we would like to note that laminar borders are not perfectly sharp but rather fluid and represent an anatomical gradient rather that an abrupt change in cell types or morphology. Accordingly, defining a “ground truth” for evaluating the performance is problematic. IB4, for example, labels the central projections of non-peptidergic sensory afferents and therefore—while predominantly labelling lamina II—neither exclusively nor completely does so. The same is true for the use of PKC*γ*, which labels a characteristic band of neurons concentrated around the transition of lamina II and III, but it is well established that some neurons escape this band. However, the fact that the great majority of the IB4- and PKC*γ*-positive pixels were mapped into the expected laminae and that similar distributions were obtained with both alignment modalities, demonstrates that the alignment of our reference maps based on the outline of the dorsal horn reliably reproduces the anatomical position of these established laminar landmarks without requiring dedicated marker channels during routine image registration.

#### 3.5.3 Evaluation of laminar cell assignment

Ultimately, the purpose of the registration workflow is not to optimize image alignment itself but to enable reproducible assignment of labeled cells to individual Rexed's laminae. Because dorsal horn neurons typically have cell body diameters of approximately 20 μm, small differences in template alignment (see range and confidence intervals mentioned in Section 3.5.1 and shown in Figure 2B) are expected to have only limited influence on laminar assignment except for neurons located immediately adjacent to laminar boundaries.

To evaluate this assumption experimentally, spinal cord sections spanning all analyzed spinal segments were immunolabeled for Paired Box Protein 2 (PAX2), a marker expressed by the majority of inhibitory interneurons distributed throughout the dorsal horn (Figure 3A and Supplementary Material for description of immunolabelling procedure) (20, 37). The five participants independently aligned the reference maps to both the brightfield and autofluorescence images before quantifying the number of PAX2-positive neurons assigned to each lamina using the plugin.

**Figure 3:**
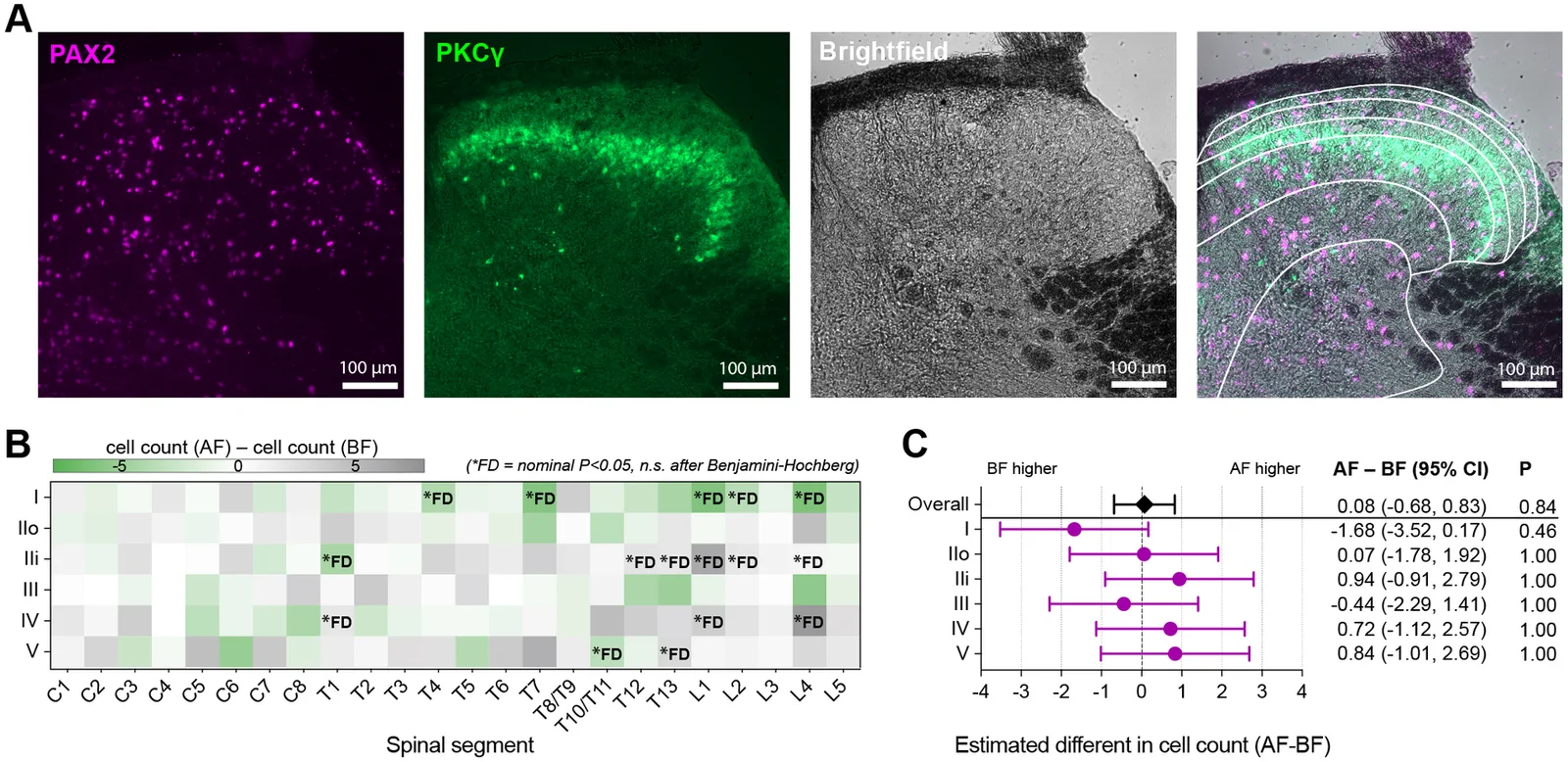
Evaluation of laminar cell assignment. **(A)** Representative image showing PAX2-positive cells in the dorsal horn (left), PKC*γ*-positive cells (second from left), a brightfield image (third from left) and a composite image of all three channels with the aligned and superimposed reference map (right). **(B)** Heatmap showing the mean differences in cell counts per lamina and spinal segment obtained after alignment to brightfield (BF) and autofluorescence (AF) images. For each lamina and segment AF and BF-derived cell counts were compared using students T-tests (*P*-values are shown in Supplementary Figure 1) followed by Benjamini-Hochberg to control for false discovery. Note, none of the differences remained significant after Benjamini-Hochberg correction (marked by *FD). **(C)** Forest plot (left) and table (right) showing the result of the mixed model analysis. Symbols show the mean difference between cell counts derived from autofluorescence- and brightfield-based alignment across all segments and users, either overall or separately for each lamina, while the horizontal lines represent the corresponding 95% confidence intervals. The confidence intervals were calculated from the uncertainty estimated by the complete model and therefore incorporate variability between users, segment-specific reference maps and repeated measurements.

Differences between the two alignment modalities were first examined separately for each spinal segment and lamina. For every matched user, segment and lamina, the cell count obtained after brightfield alignment was subtracted from the corresponding count obtained after autofluorescence alignment. All 144 comparisons, displayed as bar graphs with the individual user values superimposed, are provided in Supplementary Figure 1. The mean absolute differences between alignment modalities for each segment–lamina combination are summarized in the heat map in Figure 3B, with positive values indicating higher counts (grey) after autofluorescence alignment and negative values (green) indicating higher counts after brightfield alignment. Differences occurred in both directions without a consistent pattern. Although 16 of the 144 paired comparisons yielded nominal *P*-values below 0.05, none remained significant after correction using either the Holm or Benjamini–Hochberg procedure (Figure 3B). To assess the complete dataset in a single analysis, we additionally fitted a linear mixed-effects model with alignment modality, lamina and their interaction as fixed effects. The model accounted for repeated measurements from the same users and segments and allowed the effect of alignment modality to vary between segment-specific reference maps. The model-estimated differences are presented as a forest plot in Figure 3C. The symbols show the mean difference between autofluorescence- and brightfield-based alignment across all segments and users, either overall or separately for each lamina, while the horizontal lines represent the corresponding 95% confidence intervals. These intervals were calculated from the uncertainty estimated by the complete model and therefore incorporate variability between users, segment-specific reference maps and repeated measurements. Note, all lamina-specific 95% confidence intervals obtained from the mixed model included zero, indicating that none of the estimated cell count differences between alignment modalities was statistically distinguishable from zero. Lamina I had the widest confidence interval and the biggest estimated difference between alignment modalities and is therefore the least reliably mapped lamina. Moreover, averaged across all users, segments and laminae, the estimated difference was 0.08 cells (95% CI: −0.68 to 0.83; *P* = 0.843). The effect of alignment modality did not differ significantly among laminae (modality  ×  lamina interaction: *F*_5_,_1405_ = 1.15, *P* = 0.334), and none of the lamina-specific contrasts was significant after Holm correction.

Together, these findings demonstrate that “Dorsal Horn Mapping” provides a robust and reproducible framework for registering transverse spinal cord sections. Registration can be performed consistently by users with widely differing levels of anatomical experience, faithfully reproduces established anatomical landmarks and results in highly reproducible assignment of neuronal populations to Rexed's laminae, irrespective of whether brightfield or autofluorescence images are used for alignment.

## 4 Results

### 4.1 Representative application of the dorsal horn mapping workflow

To demonstrate the practical application of the “Dorsal Horn Mapping” framework, we analyzed the spatial distribution of calretinin (CALB2; CR)- and reelin (RELN)-expressing neurons in transverse sections of the L4 spinal dorsal horn (Figure 4A). This experiment illustrates a typical use case of the toolbox, from image registration and cell annotation to quantitative laminar analysis and visualization of pooled datasets.

**Figure 4:**
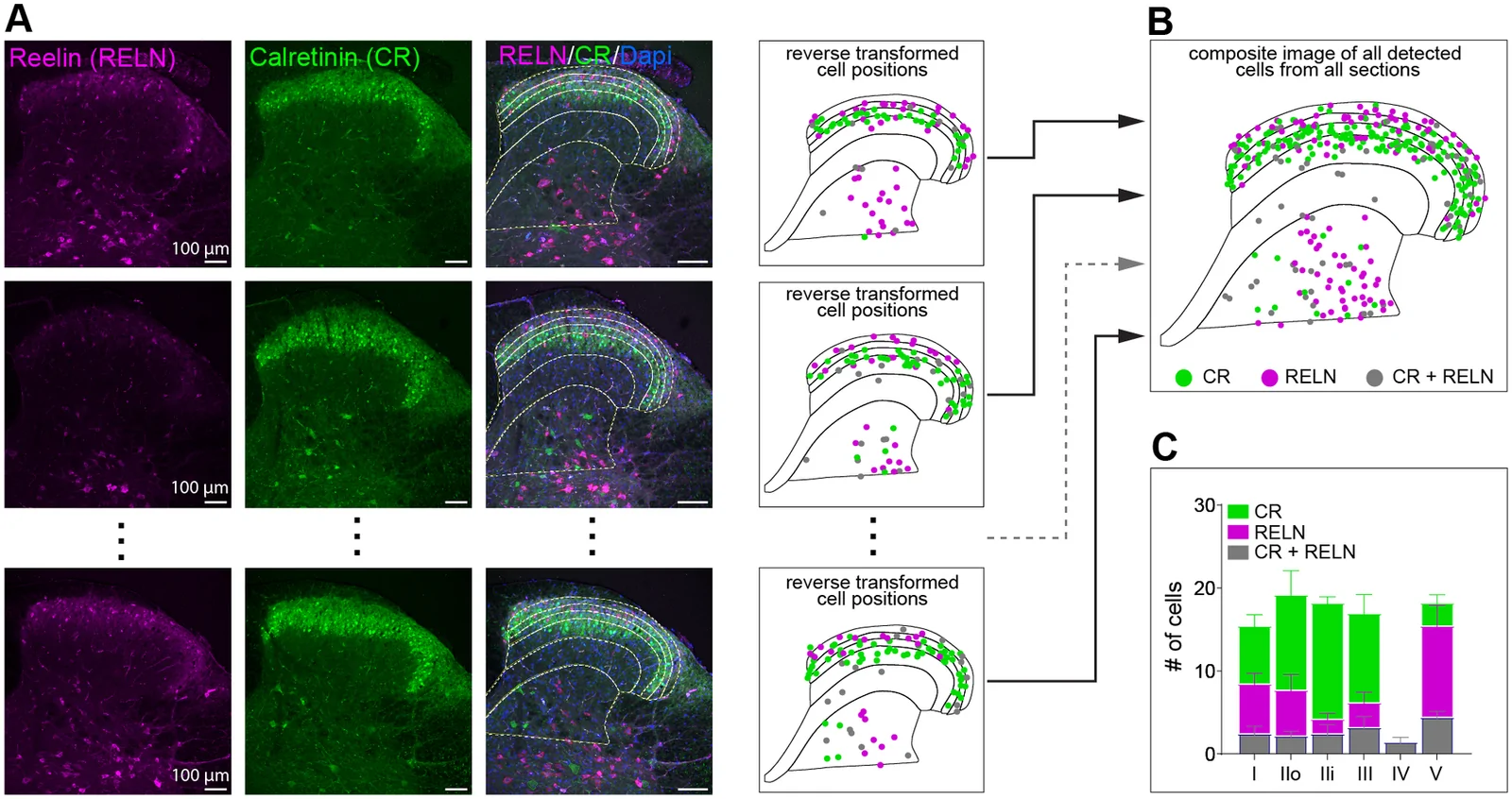
Representative application of the “Dorsal Horn Mapping” workflow. **(A)** Three example images from a series of XY sections, labelled with anti-Reelin (RELN, left), anti-Calretinin (CR, middle) and DAPI (right, show as a composite image with the other two channels) as well as graphs showing the reverse transformed positions of CR^+^/RELN^–^ (green), CR^–^/RELN^+^ (purple) and CR^+^/RELN^+^ (grey) cells from the corresponding images in the common reference map. **(B)** Composite graph showing all cells detected in the series of images (*N* = 4) in a single image. **(C)** Stacked bar graph showing the number of CR^+^/RELN^–^ (green), CR^–^/RELN^+^ (purple) and CR^+^/RELN^+^ (grey) cells per lamina created from data provided in the XLS-spreadsheets exported by the plugin.

Spinal cord sections were immunolabeled for calretinin and reelin, and positively labeled neurons were identified manually. Individual ROIs were subsequently assigned to one of three groups using the integrated grouping tool: CR^+^/RELN^−^, CR^−^/RELN^+^, or CR^+^/RELN^+^ (see Supplementary Material for description of experimental procedure). Following registration of the segment-specific L4 reference map to the outline of the dorsal horn, the plugin transformed all ROIs into normalized spatial coordinates within the common dorsal horn reference frame and assigned each neuron to its corresponding Rexed lamina. For every analyzed section, the resulting coordinates, laminar assignments, and transformation parameters were exported automatically as spreadsheet files.

The normalized coordinate data were subsequently visualized using the integrated plotting module. The “By Image” mode generated individual reference maps for each analyzed section and facilitated quality control by allowing visual inspection of every registration (Figure 4A). More importantly, the “By Segment” mode combined all neurons from all analyzed L4 sections into a single composite reference map (Figure 4B), thereby providing an integrated representation of the complete experimental dataset. In parallel, the exported coordinate tables were used to quantify the number of neurons belonging to each experimental group within the individual Rexed laminae, and the results were summarized as stacked bar graphs (Figure 4C).

This representative application highlights two key advantages of the workflow. First, transformation of cellular positions into normalized spatial coordinates enables direct comparison of spatial distributions across multiple tissue sections without the need to inspect individual images separately. Second, composite reference maps provide a transparent overview of the complete dataset by visualizing the location of every analyzed cell within a common anatomical framework. In contrast to conventional presentation of one or a few representative micrographs, this approach allows readers and reviewers to assess the full spatial distribution of labeled cells across the entire experiment while preserving quantitative information on laminar localization.

Although demonstrated here using a conventional dual-label immunohistochemical experiment, the workflow is equally applicable to other imaging modalities that generate cellular localization data, including fluorescent reporter mouse lines, *in situ* hybridization, activity-dependent labeling, viral tracing experiments, and emerging spatial transcriptomic approaches. Because the plugin works independent of the method used to identify individual cells, it can be readily integrated into existing image-analysis pipelines and provides a standardized spatial reference system for comparing diverse datasets within a common anatomical context.

## 5 Discussion

The organization of the spinal dorsal horn into Rexed's laminae provides the anatomical framework for interpreting the localization and function of neuronal populations and has therefore become deeply embedded in pain research in general and spinal cord research in particular (2–4). Whether investigating molecularly defined interneuron populations, activity-dependent labeling, viral tracing experiments, or transcriptomic datasets, the anatomical position of labeled cells is almost invariably described relative to the individual dorsal horn laminae. Despite this widespread use of laminar nomenclature, however, there is currently no broadly adopted standard for assigning experimental observations to these anatomical domains. Consequently, laboratories often rely on locally established registration strategies that differ considerably in their implementation and are frequently only incompletely described in published studies (16, 23–29). We believe that this lack of standardization represents an underappreciated source of variability that complicates direct comparison of datasets across laboratories. The framework presented here addresses this problem by introducing a simple, transparent, and reproducible workflow for laminar registration that establishes a common spatial reference system for routine analysis of transverse spinal cord sections.

Registration of spinal cord sections presents several intrinsic challenges. Histological processing inevitably introduces distortions through tissue shrinkage, compression, stretching, tearing, or partial loss of tissue, while section orientation and cutting angle introduce additional variability between preparations. At the same time, the dorsal horn lacks clearly visible anatomical landmarks that delineate all individual laminae. Lamina II can often be recognized by its translucent appearance in transmitted-light images and may be visualized using isolectin B4 labeling, whereas PKC*γ* immunoreactivity provides a useful landmark around the transition between inner lamina II and lamina III (31–33). However, these markers only identify selected anatomical regions, occupy fluorescence channels that are often required for experimental labeling, and remain dependent on staining quality, imaging conditions, and threshold selection. Likewise, estimating laminar boundaries by measuring fixed distances from the dorsal horn surface can be effective in parasagittal sections but becomes considerably more challenging in transverse sections because the curved dorsal horn surface requires measurements perpendicular to the local tissue contour rather than along a fixed image axis. Together, these factors make reproducible manual registration inherently difficult and illustrate why a standardized workflow is desirable.

Our objective was not to maximize registration accuracy at any cost, but rather to develop a framework that combines sufficient anatomical accuracy with transparency, reproducibility, and ease of implementation. This design philosophy distinguishes our “Dorsal Horn Mapping” plugin from more sophisticated atlas-registration platforms. The recently introduced SpinalJ framework represents an outstanding resource for comprehensive three-dimensional analysis of spinal cord anatomy and provides powerful tools for automated atlas registration, cell detection, and quantitative spatial analysis (34, 35). Such functionality is indispensable for large-scale anatomical studies. In contrast, the present framework was developed for the considerably more common situation in which researchers analyze relatively small numbers of transverse spinal cord sections using conventional immunohistochemistry, *in situ* hybridization, or fluorescent reporter mouse lines. For these applications, direct visual control of the registration process, straightforward integration into existing Fiji-based workflows, and minimal computational complexity are often more important than highly flexible nonlinear registration algorithms.

The deliberate use of user-guided rigid transformations and independent scaling further reflects this philosophy. More flexible affine or nonlinear image registration methods can undoubtedly achieve closer correspondence between individual tissue sections and anatomical reference atlases, particularly when combined with machine-learning-based optimization. However, increasing algorithmic flexibility also increases the risk that severely distorted tissue sections are forced into anatomically implausible registrations that may appear convincing despite substantial local inaccuracies. By restricting the registration to a limited number of intuitive transformations that remain visible to the user throughout the alignment process, the present workflow emphasizes transparency over maximal mathematical flexibility. Every transformation can be inspected visually, reproduced subsequently, and exported together with the analyzed dataset, thereby providing a complete record of the registration process.

An important conceptual aspect of the framework is the introduction of normalized spatial coordinates within a common dorsal horn reference frame. Rather than merely assigning individual cells to discrete laminae, this approach allows the complete spatial distribution of neuronal populations to be represented in a standardized anatomical coordinate system. Such normalized coordinates facilitate direct comparison of datasets obtained from different animals, experiments, and laboratories and provide a natural basis for future integration with complementary technologies. Similar standardization efforts are currently emerging in single-cell transcriptomics and spatial transcriptomics, where harmonized reference atlases have become essential for integrating datasets across laboratories (22).

Another important feature of the proposed workflow is the generation of composite reference maps. Scientific publications traditionally present one or a few representative micrographs to illustrate the anatomical distribution of labeled cells. While such images remain indispensable for assessing labeling quality and cellular morphology, they inevitably display only a small fraction of the complete dataset. Composite reference maps complement representative images by displaying the position of every analyzed cell within a standardized anatomical framework. We believe that this approach provides a substantially more transparent representation of spatial labeling data and enables readers and reviewers to evaluate the complete experimental dataset rather than relying solely on selected examples. The widespread adoption of similar visualization strategies could therefore contribute to improved reporting standards and facilitate quantitative comparison of spatial datasets across independent studies.

Several limitations of the present framework should be acknowledged. First, the reference maps are derived from a standardized anatomical atlas that was obtained from C57Bl/6 mice age P56 and therefore cannot capture all biological variability in spinal cord morphology between individual animals, developmental stages, different mouse strains, species and disease models. Second, the workflow was specifically developed for two-dimensional transverse sections and is not intended to replace comprehensive three-dimensional atlas-registration approaches. Third, the current implementation relies on manual image registration and therefore retains a user-dependent component, although our validation experiments demonstrate that this contribution has only a minor influence on downstream laminar assignment. Finally, the framework standardizes spatial registration but does not replace careful experimental design, robust cell detection, or appropriate biological controls.

Future developments may further extend the capabilities of the framework. Automated landmark detection, machine-learning-assisted registration, probabilistic assignment of cells located near laminar boundaries, and direct integration with automated cell segmentation pipelines represent promising directions for future development. Likewise, incorporation of additional anatomical templates covering other developmental stages, species, or pathological conditions would further broaden the applicability of the workflow. Because the underlying registration strategy is independent of the upstream imaging modality, these developments could readily be incorporated without fundamentally changing the analytical framework presented here.

In conclusion, “Dorsal Horn Mapping” provides a practical and accessible framework for standardized laminar registration of transverse spinal cord sections. By combining intuitive image registration with normalized spatial coordinates and transparent visualization of complete datasets, the framework establishes a common anatomical reference system that facilitates direct comparison of cellular localization across experiments and laboratories. We hope that widespread adoption of standardized spatial registration workflows—and routine publication of normalized coordinate datasets alongside microscopy images—will contribute to improved reproducibility, greater transparency, and the establishment of common reporting standards for spinal dorsal horn research.

## Data Availability

The datasets presented in this study can be found in online repositories. The names of the repository/repositories and accession number(s) can be found below: https://github.com/LucasRoettger/Dorsal-Horn-Mapping.
