# Supplementary material for: Dorsal horn mapping: a Fiji toolbox for standardized laminar registration and transparent reporting of spinal cord imaging data from adult C57Bl/6 mice
Source: Front Pain Res (Lausanne). 2026 Oct 7;7:1961590. doi: 10.3389/fpain.2026.1961590 (PMC13683651; doi:10.3389/fpain.2026.1961590)
Supplement: Supplementary file 1 [file Datasheet1.docx]

Supplementary Material

# 1 Supplementary Data

## 1.1 Animals

All experiments were conducted in accordance with the European Communities Council Directive (EU and institutional guidelines) including the ethical guidelines of ‘Protection of Animals Act’ under supervision of the ‘Animal Welfare Officers’ of University Medical Center Hamburg-Eppendorf and were approved by the local governing body (approval number …*add current valid nr*...). ARRIVE guidelines were followed. Adult male mouse C57Bl/6J (11 weeks old, n = 1) was used for sectioning the cervical to lumbar spinal cord.

## 1.2 Antibodies

The following primary and secondary antibodies were used: Rabbit anti-PKC-gamma (Proteintech, Cat# 14364-1-AP, RRID:AB_2300044, 1:500), Goat anti-Pax2 (R&D Systems, Cat# AF3364, RRID:AB_10889828, 1:200), Isolectin GS-IB4 from *Griffonia simplicifolia* Alexa Fluor™ 568 Conjugate (Invitrogen™/Thermo Fisher Scientific, Cat#I21412, 3 μg/ml), Guinea pig anti-Calretinin (Swant, Cat# CRgp7, RRID:N/A, 1:1000), Goat anti-Reelin (R&D Systems, Cat# AF3820, RRID:AB_2253745, 1:500), Alexa Fluor 555 Donkey Anti-Rabbit IgG H&L (Abcam Cat# ab150074, RRID:AB_2636997 1:500), Alexa Fluor 555 Donkey Anti-Goat IgG H&L (Abcam Cat# ab150130, RRID:AB_2927775, 1:500), Alexa Fluor 647-AffiniPure Donkey Anti-Guinea Pig IgG (Jackson ImmunoResearch Labs Cat# 706-605-148, RRID:AB_2340476, 1:500), Alexa Fluor 647 conjugated donkey anti-Goat IgG (Invitrogen™/Thermo Fisher Scientific, Cat# A32849, RRID:AB_2762840, 1:500)

## 1.3 Tissue processing

For spinal cord slice preparation, mice were sacrificed by placing them in a CO_2_-filled chamber for 2–4 min, followed by cervical dislocation. Spinal column was dissected and transferred to the dissection chamber, filled with the ice-cold (~4°C) dissection solution consisting of (in mM): 95 NaCl, 1.8 KCl, 7 MgSO_4_, 0.5 CaCl_2_, 1.2 KH_2_PO_4_, 26NaHCO_3_, 25D-glucose, and 50 sucrose) saturated with carbogen (95% O_2_,5% CO_2_). The spinal column was fixed by insect pins to the Sylgard bottom layer of the dissection chamber under the binocular microscope Olympus SZ61. To prevent damage of the dorsal part, ventral laminectomy was used to expose and dissect the spinal cord from the spinal canal. Isolated spinal cord was carefully free from the *Dura mater*. Ventral roots were removed, while dorsal roots were kept intact for subsequent identification of spinal cord segments before segmentation. The spinal cord was then fixed in Zamboni’s fixative (phosphate-buffered (PBS) picric acid-formaldehyde solution) overnight. The fixed spinal cord was cryoprotected subsequently in 15% and 30% sucrose (in 1M PBS) solution at 4°C until sunk down (usually 24-48h). Cryoprotected spinal cord was then under the binocular microscope segmented by scalpel into two cervical, three thoracal and two lumbar (and one sacral) specimens. Each specimen was in a defined rostro-caudal and dorso-vetnral direction embedded in a plastic chamber (Tissue-Tek® Cryomold, 10 x 10 x 5 mm, Sakura Finetek Europe) filled with optimum cutting temperature compound (Tissue-Tek® O.C.T. Compound, Sakura Finetek Europe) and frozen. Spinal cord specimens were cut on the cryostat (Leica CM3000, Germany) into 30 μm consecutive transversal cryo-sections in rostro-caudal direction and mounted onto microscope slides (Superfrost PLUS Adhesion slides; Epredia, Gerhard Menzel GmbH, Germany). For each spinal cord segment, 2 slices in 5 series were cut.

## 1.4 Immunohistochemistry

After drying at RT for 1 h, sections were incubated with 50 mM Glycine in PBS for 30 min, washed 4 x 10 min with PBS, permeabilised 1 h with 0.2% Triton-X100 in PBS (0.2 % PBST), blocked for 1 h in blocking solution (10% normal donkey serum (NDS) in 0.2% PBST) and then incubated in primary antibodies mixture overnight at 4°C. Primary antibodies were diluted in the blocking solution (10% NDS in 0.2% PBST). The next day, slides were washed 5 x 10 min in PBS and incubated with secondary antibody mix in blocking solution (possibly with IB4) overnight at 4°C. Finally, slides were washed 5 x 10 min in PBS. After the last wash step, liquid was carefully removed by touching the edges of the slides against a paper towel and 3-4 drops of mounting media (ProLong™ Diamond Antifade Mountant with DAPI, Invitrogen, Cat# P36962) was applied to the specimen and by tilting the slide, it was equally spread it on the spinal cord slices. 2-3 minutes waiting time was then used for optimal DAPI penetration and labelling of the specimen before applying a cover glass and transparent nail polish to prevent its movement and drying. Samples were stored in the dark at 4°C before imaging.

## 1.5 Imaging

All images were acquired on a Zeiss Axio Observer with 10x objective (A-Plan Ph1) and a CCD AxioCam HRm 1.4MP monochrome camera. The images where then directly imported into FIJI and only the brightness and contrast were adjusted manually to the user’s preferences. To segment the PAX2^+^ cells, a self-written algorithm was used, and the results were manually approved by LR.

## 1.6 Statistics

All statistical analyses were performed with custom MATLAB scripts. The bar graphs were generated, and t-tests were performed in GraphPad Prism 10 ([www.graphpad.com](http://www.graphpad.com)).

# 2 Supplementary Figures and Tables

## 2.1 Supplementary Tables

|  | **Map position X (px)** | | | | | **Map position Y (px)** | | | | | **Rotation (deg)** | | | | | **Scale factor X** | | | | | **Scale factor Y** | | | | |
| --- | --- | --- | --- | --- | --- | --- | --- | --- | --- | --- | --- | --- | --- | --- | --- | --- | --- | --- | --- | --- | --- | --- | --- | --- | --- |
|  | **User 1** | **User 2** | **User 3** | **User 4** | **User 5** | **User 1** | **User 2** | **User 3** | **User 4** | **User 5** | **User 1** | **User 2** | **User 3** | **User 4** | **User 5** | **User 1** | **User 2** | **User 3** | **User 4** | **User 5** | **User 1** | **User 2** | **User 3** | **User 4** | **User 5** |
| C1 | 789 | 748 | 710 | 688 | 778 | 438 | 402 | 404 | 362 | 416 | 55 | 50 | 46 | 45 | 55 | 1.04 | 1.1 | 1.12 | 1.2 | 1.08 | 1.06 | 1.1 | 1.08 | 1.1 | 1.04 |
| C2 | 35 | 563 | 703 | 435 | 657 | 396 | 536 | 560 | 436 | 464 | 15 | 42 | 62 | 40 | 60 | 1.7 | 0.86 | 0.68 | 1.2 | 0.96 | 1.9 | 1.2 | 1.02 | 1.1 | 1.02 |
| C3 | 573 | 593 | 589 | 613 | 611 | 470 | 473 | 468 | 464 | 470 | 42 | 45 | 45 | 46 | 46 | 1.06 | 1.1 | 1.02 | 1 | 1.06 | 1 | 1 | 1 | 1 | 1 |
| C4 | 655 | 599 | 579 | 683 | 633 | 347 | 387 | 367 | 367 | 355 | 74 | 70 | 68 | 76 | 75 | 1.24 | 1.1 | 1.12 | 1.16 | 1.1 | 1.02 | 1.2 | 1.18 | 1 | 1.1 |
| C5 | 647 | 588 | 679 | 631 | 665 | 369 | 409 | 341 | 351 | 339 | 75 | 65 | 80 | 73 | 78 | 1.1 | 1.2 | 1.1 | 1.18 | 1.1 | 1.5 | 1.6 | 1.4 | 1.44 | 1.42 |
| C6 | 754 | 614 | 706 | 674 | 676 | 388 | 408 | 338 | 348 | 367 | 75 | 60 | 74 | 70 | 70 | 1.2 | 1.2 | 1.16 | 1.3 | 1.2 | 1 | 1.2 | 0.96 | 1 | 1.04 |
| C7 | 571 | 452 | 463 | 351 | 483 | 371 | 271 | 289 | 311 | 316 | 95 | 80 | 83 | 70 | 85 | 1 | 1.2 | 1.1 | 1.2 | 1.1 | 0.9 | 1 | 1.04 | 1.2 | 1.02 |
| C8 | 712 | 752 | 798 | 728 | 738 | 302 | 342 | 356 | 324 | 352 | 95 | 100 | 108 | 97 | 100 | 1.1 | 1 | 0.98 | 1.02 | 0.98 | 1.2 | 1.1 | 1 | 1.08 | 1.14 |
| T1 | 565 | 587 | 542 | 507 | 579 | 212 | 236 | 198 | 230 | 222 | 125 | 127 | 120 | 116 | 129 | 1.2 | 1.2 | 1.22 | 1.16 | 1.2 | 1.4 | 1.32 | 1.4 | 1.42 | 1.42 |
| T2 | 854 | 848 | 798 | 762 | 774 | 465 | 485 | 435 | 447 | 437 | 120 | 113 | 111 | 105 | 113 | 0.8 | 0.8 | 0.8 | 0.76 | 0.84 | 0.9 | 0.8 | 0.94 | 0.96 | 1 |
| T3 | 738 | 714 | 712 | 702 | 684 | 572 | 562 | 552 | 542 | 504 | 135 | 132 | 132 | 125 | 137 | 0.7 | 0.74 | 0.72 | 0.64 | 0.68 | 0.7 | 0.8 | 0.82 | 0.88 | 0.84 |
| T4 | 736 | 636 | 712 | 650 | 652 | 390 | 390 | 382 | 392 | 382 | 100 | 90 | 98 | 84 | 92 | 0.9 | 0.9 | 0.92 | 0.94 | 0.92 | 0.7 | 0.9 | 0.74 | 0.84 | 0.84 |
| T5 | 845 | 817 | 847 | 826 | 864 | 440 | 416 | 448 | 446 | 448 | 120 | 131 | 123 | 115 | 123 | 0.7 | 0.76 | 0.74 | 0.68 | 0.74 | 0.7 | 0.8 | 0.7 | 0.72 | 0.66 |
| T6 | 429 | 608 | 507 | 510 | 587 | 468 | 447 | 421 | 452 | 458 | 60 | 100 | 77 | 75 | 98 | 1 | 0.8 | 0.94 | 0.9 | 0.86 | 1.1 | 1 | 1.04 | 1 | 1.02 |
| T7 | 571 | 691 | 697 | 531 | 659 | 429 | 408 | 399 | 509 | 401 | 40 | 65 | 63 | 30 | 53 | 1 | 0.9 | 0.92 | 1 | 0.9 | 1.2 | 1.1 | 1 | 1.4 | 1 |
| T8_T9 | 660 | 730 | 742 | 768 | 734 | 380 | 406 | 376 | 379 | 374 | 65 | 81 | 79 | 80 | 82 | 1 | 0.94 | 0.96 | 0.94 | 1 | 1 | 1 | 0.88 | 0.76 | 0.92 |
| T10_T11 | 829 | 828 | 875 | 881 | 881 | 492 | 531 | 489 | 522 | 498 | 45 | 70 | 62 | 62 | 59 | 0.8 | 0.8 | 0.72 | 0.76 | 0.78 | 0.8 | 0.9 | 0.76 | 0.8 | 0.72 |
| T12 | 660 | 702 | 713 | 701 | 703 | 410 | 455 | 444 | 450 | 436 | 95 | 105 | 105 | 110 | 104 | 0.9 | 0.9 | 0.8 | 0.8 | 0.86 | 1.1 | 1 | 0.98 | 1 | 1 |
| T13 | 521 | 581 | 523 | 561 | 533 | 361 | 381 | 375 | 401 | 377 | 80 | 90 | 81 | 85 | 81 | 1 | 0.9 | 0.94 | 0.9 | 0.96 | 1.2 | 1.1 | 1.18 | 1.1 | 1.16 |
| L1 | 776 | 782 | 798 | 772 | 812 | 407 | 417 | 413 | 429 | 409 | 107 | 106 | 107 | 104 | 108 | 0.78 | 0.72 | 0.76 | 0.72 | 0.76 | 0.8 | 0.8 | 0.74 | 0.78 | 0.7 |
| L2 | 870 | 870 | 866 | 884 | 882 | 417 | 417 | 405 | 419 | 415 | 115 | 120 | 118 | 119 | 118 | 0.8 | 0.8 | 0.78 | 0.78 | 0.8 | 0.8 | 0.8 | 0.8 | 0.74 | 0.76 |
| L3 | 772 | 822 | 810 | 814 | 806 | 432 | 466 | 442 | 456 | 454 | 80 | 78 | 80 | 74 | 78 | 0.8 | 0.8 | 0.74 | 0.74 | 0.78 | 0.9 | 0.82 | 0.8 | 0.78 | 0.8 |
| L4 | 735 | 815 | 794 | 794 | 828 | 506 | 486 | 496 | 506 | 496 | 65 | 80 | 77 | 75 | 81 | 0.8 | 0.72 | 0.72 | 0.7 | 0.76 | 0.9 | 0.8 | 0.78 | 0.8 | 0.74 |
| L5 | 518 | 468 | 512 | 526 | 514 | 457 | 469 | 457 | 463 | 465 | 75 | 76 | 75 | 73 | 76 | 1 | 0.94 | 0.9 | 0.9 | 0.92 | 0.9 | 1 | 0.9 | 0.9 | 0.94 |

**Supplementary Table1**. Transformation parameters for brightfield aligned reference maps for all users.

|  | **Map position X (px)** | | | | | **Map position Y (px)** | | | | | **Rotation (deg)** | | | | | **Scale factor X** | | | | | **Scale factor Y** | | | | |
| --- | --- | --- | --- | --- | --- | --- | --- | --- | --- | --- | --- | --- | --- | --- | --- | --- | --- | --- | --- | --- | --- | --- | --- | --- | --- |
|  | User 1 | User 2 | User 3 | User 4 | User 5 | User 1 | User 2 | User 3 | User 4 | User 5 | User 1 | User 2 | User 3 | User 4 | User 5 | User 1 | User 2 | User 3 | User 4 | User 5 | User 1 | User 2 | User 3 | User 4 | User 5 |
| C1 | 748 | 828 | 722 | 708 | 762 | 422 | 422 | 410 | 462 | 434 | 50 | 65 | 50 | 55 | 58 | 1.1 | 1 | 1.1 | 1.2 | 1.08 | 1.1 | 1 | 1.1 | 1.2 | 1.1 |
| C2 | 155 | 615 | 718 | 555 | 668 | 576 | 476 | 560 | 476 | 496 | 0 | 55 | 69 | 45 | 57 | 1.4 | 0.9 | 0.76 | 1 | 0.9 | 2 | 1.1 | 0.9 | 1.2 | 1.02 |
| C3 | 493 | 593 | 603 | 553 | 591 | 572 | 472 | 478 | 492 | 482 | 25 | 45 | 45 | 50 | 50 | 1.1 | 1.1 | 1.12 | 1.1 | 1.02 | 1.2 | 1 | 1 | 1.2 | 1.12 |
| C4 | 639 | 578 | 587 | 599 | 617 | 367 | 347 | 347 | 387 | 369 | 80 | 80 | 77 | 80 | 75 | 1.2 | 1.1 | 1.1 | 1.3 | 1.12 | 1.1 | 1.2 | 1.18 | 1.2 | 1.16 |
| C5 | 546 | 607 | 703 | 766 | 746 | 329 | 369 | 343 | 349 | 349 | 85 | 70 | 84 | 105 | 90 | 1.3 | 1.1 | 1.06 | 1.2 | 1.1 | 1.8 | 1.5 | 1.36 | 1.4 | 1.3 |
| C6 | 714 | 574 | 722 | 713 | 652 | 388 | 388 | 366 | 388 | 367 | 85 | 55 | 82 | 85 | 70 | 1.3 | 1.2 | 1.16 | 1.2 | 1.2 | 1 | 1.2 | 0.98 | 1 | 1.08 |
| C7 | 571 | 505 | 483 | 431 | 494 | 411 | 321 | 315 | 311 | 339 | 105 | 90 | 92 | 85 | 91 | 1.1 | 1 | 1.14 | 1.2 | 1.12 | 0.9 | 1.02 | 1.02 | 1.1 | 1.02 |
| C8 | 752 | 664 | 818 | 532 | 704 | 322 | 286 | 386 | 342 | 336 | 100 | 86 | 105 | 85 | 98 | 1.1 | 1.12 | 0.88 | 1.1 | 0.96 | 1.1 | 1.2 | 0.92 | 1.5 | 1.24 |
| T1 | 505 | 585 | 571 | 545 | 569 | 192 | 232 | 246 | 252 | 234 | 130 | 125 | 125 | 115 | 125 | 1.4 | 1.2 | 1.26 | 1.2 | 1.26 | 1.6 | 1.3 | 1.34 | 1.3 | 1.34 |
| T2 | 798 | 815 | 804 | 694 | 782 | 457 | 465 | 455 | 425 | 449 | 122 | 115 | 119 | 105 | 120 | 0.9 | 0.8 | 0.82 | 0.8 | 0.86 | 0.98 | 0.9 | 0.92 | 1.2 | 1 |
| T3 | 698 | 678 | 738 | 696 | 677 | 532 | 592 | 538 | 571 | 499 | 140 | 135 | 134 | 133 | 135 | 0.7 | 0.8 | 0.64 | 0.76 | 0.7 | 0.9 | 0.8 | 0.8 | 0.82 | 0.84 |
| T4 | 716 | 657 | 744 | 648 | 654 | 430 | 370 | 426 | 412 | 386 | 110 | 100 | 101 | 100 | 98 | 1 | 0.9 | 0.88 | 1.04 | 0.94 | 0.8 | 0.9 | 0.7 | 0.9 | 0.88 |
| T5 | 805 | 808 | 868 | 765 | 864 | 440 | 430 | 458 | 400 | 456 | 125 | 127 | 122 | 115 | 120 | 0.8 | 0.78 | 0.7 | 0.7 | 0.68 | 0.8 | 0.8 | 0.66 | 0.9 | 0.7 |
| T6 | 509 | 575 | 619 | 451 | 579 | 428 | 480 | 442 | 464 | 464 | 85 | 104 | 93 | 78 | 95 | 1 | 0.86 | 0.78 | 1 | 0.9 | 1.1 | 1.1 | 0.88 | 1.12 | 1.02 |
| T7 | 671 | 731 | 709 | 731 | 711 | 429 | 429 | 390 | 449 | 409 | 60 | 65 | 63 | 70 | 69 | 1.1 | 1 | 0.88 | 1 | 0.94 | 1.1 | 1 | 0.98 | 1 | 1 |
| T8_T9 | 700 | 700 | 744 | 780 | 740 | 379 | 380 | 371 | 420 | 403 | 80 | 85 | 85 | 90 | 86 | 1.1 | 1 | 0.92 | 1 | 0.96 | 1 | 1 | 0.84 | 0.8 | 0.9 |
| T10_T11 | 868 | 849 | 859 | 869 | 849 | 552 | 531 | 484 | 591 | 514 | 65 | 65 | 62 | 65 | 64 | 0.9 | 0.8 | 0.72 | 0.9 | 0.76 | 0.8 | 0.8 | 0.72 | 0.8 | 0.78 |
| T12 | 720 | 720 | 718 | 700 | 704 | 489 | 490 | 452 | 469 | 466 | 105 | 105 | 103 | 105 | 105 | 0.9 | 0.9 | 0.82 | 0.9 | 0.9 | 1 | 0.9 | 0.96 | 1 | 1.02 |
| T13 | 582 | 581 | 583 | 589 | 589 | 421 | 401 | 377 | 395 | 397 | 95 | 100 | 92 | 91 | 94 | 1 | 1 | 0.94 | 0.92 | 0.94 | 1.1 | 1.1 | 1.06 | 1.04 | 1.12 |
| L1 | 772 | 752 | 808 | 752 | 822 | 459 | 419 | 419 | 459 | 425 | 115 | 105 | 112 | 105 | 110 | 0.9 | 0.8 | 0.76 | 0.8 | 0.78 | 0.8 | 0.9 | 0.74 | 0.9 | 0.74 |
| L2 | 830 | 830 | 896 | 870 | 904 | 417 | 413 | 417 | 417 | 421 | 120 | 120 | 119 | 120 | 122 | 0.9 | 0.84 | 0.78 | 0.8 | 0.8 | 0.9 | 0.9 | 0.76 | 0.8 | 0.78 |
| L3 | 773 | 826 | 856 | 772 | 770 | 472 | 470 | 456 | 492 | 448 | 85 | 80 | 82 | 80 | 86 | 0.9 | 0.8 | 0.78 | 0.8 | 0.8 | 0.9 | 0.8 | 0.74 | 0.9 | 0.92 |
| L4 | 754 | 814 | 856 | 802 | 832 | 486 | 526 | 482 | 518 | 510 | 80 | 85 | 83 | 80 | 82 | 0.9 | 0.8 | 0.68 | 0.8 | 0.8 | 0.9 | 0.8 | 0.7 | 0.8 | 0.76 |
| L5 | 558 | 498 | 538 | 518 | 546 | 457 | 477 | 477 | 457 | 459 | 85 | 75 | 76 | 75 | 78 | 1 | 0.9 | 0.9 | 0.9 | 0.9 | 0.9 | 1 | 0.84 | 0.9 | 0.9 |

**Supplementary Table2**. Transformation parameters for autofluorescence aligned reference maps for all users.

## 2.3 Supplementary Figures

**Supplementary Figure 1.** **(A)** Comparisons of the absolute number detected cells in the indicated spinal segments and laminae (mean + SEM) in brightfield- (white) and autofluorescence- (purple) aligned reference maps (unpaired t-tests, p-values are shown above the graph).
